# A scoping review and expert consensus on digital determinants of health

**DOI:** 10.2471/BLT.24.292057

**Published:** 2024-10-29

**Authors:** Robin van Kessel, Laure-Elise Seghers, Michael Anderson, Nienke M Schutte, Giovanni Monti, Madeleine Haig, Jelena Schmidt, George Wharton, Andres Roman-Urrestarazu, Blanca Larrain, Yoann Sapanel, Louisa Stüwe, Agathe Bourbonneux, Junghee Yoon, Mangyeong Lee, Ivana Paccoud, Liyousew Borga, Njide Ndili, Eric Sutherland, Marelize Görgens, Eva Weicken, Megan Coder, Heimar de Fatima Marin, Elena Val, Maria Cristina Profili, Monika Kosinska, Christine Elisabeth Browne, Alvin Marcelo, Smisha Agarwal, Monique F. Mrazek, Hani Eskandar, Roman Chestnov, Marina Smelyanskaya, Karin Källander, Stefan Buttigieg, Kirthi Ramesh, Louise Holly, Andrzej Rys, Natasha Azzopardi-Muscat, Jerome de Barros, Yuri Quintana, Antonio Spina, Adnan A Hyder, Alain Labrique, Maged N Kamel Boulos, Wen Chen, Anurag Agrawal, Juhee Cho, Jochen Klucken, Barbara Prainsack, Ran Balicer, Ilona Kickbusch, David Novillo-Ortiz, Elias Mossialos

**Affiliations:** a LSE Health, Department of Health Policy, London School of Economics and Political Science, Houghton Street, London, WC2A 2AE, London, England.; b Innovation in Health Information Systems Unit, Sciensano, Brussels, Belgium.; c Department of International Health, Maastricht University, Maastricht, Kingdom of the Netherlands.; d Department of Psychiatry, University of Cambridge, Cambridge, England.; e Institute of Digital Medicine, National University of Singapore, Singapore.; f Digital Health Delegation for Digital Health, Ministry of Labour, Health and Solidarities, Paris, France.; g Department of Clinical Research Design and Evaluation, Sungkyunkwan University, Seoul, Republic of Korea.; h Luxembourg Centre for Systems Biomedicine, Université du Luxembourg, Belvaux, Luxembourg.; i PharmAccess Foundation Nigeria, Lagos, Nigeria.; j Paris, France.; k Health, Nutrition and Population Global Practice, World Bank Group, Washington DC, United States of America (USA).; l Fraunhofer Institute for Telecommunications, Heinrich Hertz Institut, Berlin, Germany.; m Digital Medicine Society, Boston, USA.; n Department of Biomedical and Data Science, Yale University School of Medicine, New Haven, USA.; o Migration Health Division, International Organization for Migration Regional Office for the European Economic Area, the EU and NATO, Brussels, Belgium.; p Department of Social Determinants of Health, World Health Organization, Geneva, Switzerland.; q WHO European Venice Office for Investment for Health and Development, Venice, Italy.; r Medical Informatics Unit, University of the Philippines, Manila, Philippines.; s Department of International Health, The Johns Hopkins University Bloomberg School of Public Health, Baltimore, USA.; t International Finance Corporation, World Bank Group, Washington DC, USA.; u Digital Services Division, International Telecommunications Union, Geneva, Switzerland.; v HIV and Health Group, United Nations Development Programme Europe and Central Asia, Istanbul, Türkiye.; w United Nations Children’s Fund, New York, USA.; x Ministry for Health and Active Ageing, Valletta, Malta.; y Asian Development Bank, Manila, Philippines.; z Digital Transformations for Health Lab, Geneva, Switzerland.; aa Health Systems, Medical Products and Innovation, European Commission, Brussels, Belgium.; ab Division of Country Health Policies and Systems, World Health Organization Regional Office for Europe, Copenhagen, Denmark.; ac Directorate General For Health and Food Safety, European Commission, Brussels, Belgium.; ad Department of Medicine, Beth Israel Deaconess Medical Center, Boston, USA.; ae Centre for Health and Healthcare, World Economic Forum, Geneva, Switzerland.; af Milken Institute School of Public Health, George Washington University, Washington DC, USA.; ag Department of Digital Health and Innovation, World Health Organization, Geneva, Switzerland.; ah Information Management School, Sun Yat-sen University, Guangzhou, China.; ai School of Public Health, Fudan University, Shanghai, China.; aj Trivedi School of Biosciences, Ashoka University, Sonepat, India.; ak Department of Political Science and Research Platform Governance of Digital Practices, University of Vienna, Vienna, Austria.; al Clalit Research Institute, Tel Aviv, Israel.

## Abstract

**Objective:**

To map how social, commercial, political and digital determinants of health have changed or emerged during the recent digital transformation of society and to identify priority areas for policy action.

**Methods:**

We systematically searched MEDLINE, Embase and Web of Science on 24 September 2023, to identify eligible reviews published in 2018 and later. To ensure we included the most recent literature, we supplemented our review with non-systematic searches in PubMed® and Google Scholar, along with records identified by subject matter experts. Using thematic analysis, we clustered the extracted data into five societal domains affected by digitalization. The clustering also informed a novel framework, which the authors and contributors reviewed for comprehensiveness and accuracy. Using a two-round consensus process, we rated the identified determinants into high, moderate and low urgency for policy actions.

**Findings:**

We identified 13 804 records, of which 204 met the inclusion criteria. A total of 127 health determinants were found to have emerged or changed during the digital transformation of society (37 digital, 33 social, 33 commercial and economic and 24 political determinants). Of these, 30 determinants (23.6%) were considered particularly urgent for policy action.

**Conclusion:**

This review offers a comprehensive overview of health determinants across digital, social, commercial and economic, and political domains, highlighting how policy decisions, individual behaviours and broader factors influence health by digitalization. The findings deepen our understanding of how health outcomes manifest within a digital ecosystem and inform strategies for addressing the complex and evolving networks of health determinants.

## Résumé

**Objectif:**

Caractériser la manière dont les déterminants sociaux, commerciaux, politiques et numériques ont changé ou sont apparus au cours de la récente transformation numérique de la société, et identifier les domaines prioritaires pour l'adoption de mesures concrètes.

**Méthodes:**

Nous avons passé au crible MEDLINE, Embase et Web of Science le 24 septembre 2023, afin de recenser les revues éligibles publiées à partir de 2018. Pour nous assurer d'inclure la littérature la plus récente, nous avons complété notre revue par des recherches non systématiques effectuées dans PubMed® et Google Scholar, ainsi que par des documents identifiés par des experts en la matière. À l'aide d'une analyse thématique, nous avons réparti les données extraites entre cinq domaines sociétaux concernés par la numérisation. Ce regroupement a également servi à établir un nouveau cadre, que les auteurs et contributeurs ont examiné pour en évaluer la précision et l'exhaustivité. Enfin, grâce à un processus de consensus en deux phases, nous avons défini le degré d'urgence des mesures politiques pour les déterminants identifiés: élevé, modéré et faible.

**Résultats:**

Nous avons recensé 13 804 documents, dont 204 correspondaient aux critères d'inclusion. Au total, 127 déterminants de santé ont été considérés comme étant apparus ou ayant changé durant la transformation numérique de la société (37 déterminants numériques, 33 déterminants sociaux, 33 déterminants commerciaux et économiques, et 24 déterminants politiques). Parmi eux, 30 déterminants (23,6%) ont été jugés particulièrement urgents en termes d'intervention politique.

**Conclusion:**

Cette revue offre une vue globale des déterminants de santé dans les domaines du numérique, du social, du commercial et de l'économique ainsi que du politique, illustrant l'impact qu'exercent les décisions politiques, les comportements individuels et d'autres facteurs plus larges sur la santé par le biais de la numérisation. Les conclusions obtenues nous permettent de mieux comprendre comment les résultats en matière de santé se présentent dans un écosystème numérique, et alimentent les stratégies visant à appréhender les réseaux des déterminants de santé, des réseaux complexes et en constante évolution.

## Resumen

**Objetivo:**

Analizar cómo han cambiado o surgido determinantes sociales, comerciales, políticos y digitales de la salud durante la reciente transformación digital de la sociedad e identificar áreas prioritarias de acción normativa.

**Métodos:**

Se realizaron búsquedas sistemáticas en MEDLINE, Embase y Web of Science el 24 de septiembre de 2023, para identificar revisiones elegibles publicadas en 2018 y posteriormente. Para asegurar la inclusión de la literatura más reciente, se complementó la revisión con búsquedas no sistemáticas en PubMed® y Google Scholar, junto con registros identificados por expertos en la materia. Mediante un análisis temático, se agruparon los datos extraídos en cinco áreas sociales afectadas por la digitalización. La agrupación también sirvió de base para un nuevo marco, cuya exhaustividad y precisión revisaron los autores y colaboradores. Mediante un proceso de consenso en dos rondas, se clasificaron los determinantes identificados en alta, moderada y baja urgencia para la adopción de acciones normativas.

**Resultados:**

Se identificaron 13 804 registros, de los que 204 cumplían los criterios de inclusión. Se descubrió que un total de 127 determinantes de la salud habían surgido o cambiado durante la transformación digital de la sociedad (37 determinantes digitales, 33 sociales, 33 comerciales y económicos y 24 políticos). De ellos, 30 determinantes (23,6%) se consideraron de especial urgencia para la acción normativa.

**Conclusión:**

Esta revisión ofrece una visión global de los determinantes de la salud en las áreas digital, social, comercial y económica, y política, y destaca cómo las decisiones políticas, los comportamientos individuales y otros factores más amplios influyen en la salud a través de la digitalización. Los resultados profundizan el conocimiento de cómo se manifiestan los resultados sanitarios en un ecosistema digital e informan de las estrategias para abordar las complejas y cambiantes redes de los determinantes de la salud.

## ملخص

**الغرض:**

تخطيط كيفية ظهور المحددات الاجتماعية، والتجارية، والسياسية، والرقمية، للصحة أو ظهور تغير فيها، أثناء التحول الرقمي الأخير للمجتمع، وكذلك تحديد المجالات ذات الأولوية للعمل السياسي.

**الطريقة:**

لقد قمنا بالبحث بشكل منهجي في MEDLINE، **و **Embase، **و **Web of Science في 24 سبتمبر/أيلول 2023، لتحديد المراجعات المؤهلة المنشورة في عام 2018 وما بعده. للتأكد من تضمين أحدث المنشورات، قمنا باستكمال المراجعات لدينا بعمليات بحث غير منهجية في قواعد البيانات PubMed **® **‎، **و **Google Scholar، جنبًا إلى جنب مع السجلات التي حددها الخبراء في هذا الصدد. باستخدام التحليل الموضوعي، قمنا بتجميع البيانات المستخرجة في خمسة مجالات مجتمعية خضعت للرقمنة. ونتج عن التجميع إطار عمل جديد، راجعه المؤلفون والمساهمون بهدف تحقيق الشمول والدقة. باستخدام عملية إجماع من جولتين، قمنا بتقييم المحددات المعروفة إلى عالية الضرورة، ومتوسطة الضرورة، ومنخفضة الضرورة لتنفيذ إجراءات السياسة **. **

**النتائج:**

حدد بحثنا 13804 سجلاً، منها 204 سجلاً حققت معايير الاشتمال. واكتشفنا أن إجمالي 127 من محددات الصحة قد ظهرت أو تغيرت أثناء التحول الرقمي للمجتمع، (37 محددًا رقميًا، و33 محددًا اجتماعيًا، و33 محددًا تجاريًا واقتصاديًا، و24 محددًا سياسيًا). ومن بين هذه المحددات، تم اعتبار 30 محددًا (%23.6) عالية ضرورية بشكل خاص لتنفيذ إجراءات السياسة.

**الاستنتاج:**

تقدم هذه المراجعة نظرة عامة شاملة لمحددات الصحة عبر المجالات الرقمية، والاجتماعية، والتجارية، والاقتصادية، والسياسية، مع التركيز على كيفية تأثير القرارات السياسية، والسلوكيات الفردية، والعوامل الأوسع نطاقًا على الصحة من خلال الرقمنة. وتعمق النتائج فهمنا لكيفية ظهور النواتج الصحية داخل النظام البيئي الرقمي، وتقديمها لاستراتيجيات للتعامل مع الشبكات المعقدة والمتطورة لمحددات الصحة.

## 摘要

**目的:**

旨在了解在最近的社会数字化转型过程中，健康的社会、商业、政治和数字决定因素发生了怎样的变化或出现了哪些新的决定因素，并确定政策行动的优先领域。

**方法:**

我们已于 2023 年 9 月 24 日系统地检索了 MEDLINE、Embase 和 Web of Science 数据库，以找出在 2018 年及之后发表的符合条件的综述文献。为确保我们纳入了最新的文献资料，通过在 PubMed^® ^和 Google Scholar 中进行非系统性检索和利用主题专家确定的记录，我们进一步补充了综述文献。通过主题分析，我们使用聚类算法将提取的数据分为五个受数字化影响的社会领域。聚类算法还为我们提供了一个新的框架，即作者和撰稿人有对其全面性和准确性进行审查的文献资料。经历两轮共识过程后，我们将最终确定的决定因素分为紧急程度高、紧急程度中等和紧急程度低三类以用于确定政策行动的优先级。

**结果:**

我们检索到 13,804 条记录，其中有 204 条符合纳入标准。我们发现，在社会的数字化转型过程中出现或发生了变化的健康决定因素共有 127 个（37 个数字决定因素、33 个社会决定因素、33 个商业和经济决定因素以及 24 个政治决定因素）。 其中，又有 30 个决定因素（占 23.6%）被认为特别迫切地需要采取政策行动。

**结论:**

本综述全面概述了数字、社会、商业和经济以及政治领域的健康决定因素，重点介绍了政策决策、个人行为和更广泛的因素如何通过数字化影响健康。这些研究结果加深了我们对健康结果如何在数字生态系统中表现的理解，并为制定策略以解决复杂和不断发展的健康决定因素网络提供了依据。

## Резюме

**Цель:**

Определить, как изменились или какие появились социальные, коммерческие, политические и цифровые детерминанты здоровья в ходе недавней цифровой трансформации общества, и выявить приоритетные области для принятия мер.

**Методы:**

24 сентября 2023 года был проведен систематический поиск в базах данных MEDLINE, Embase и Web of Science с целью выявления соответствующих требованиям обзоров, опубликованных в 2018 году и позднее. Чтобы убедиться в том, что в обзор включена самая свежая литература, в дополнение было проведено несистематическое исследование в базах данных PubMed® и Google Scholar, а также использованы данные, полученные от экспертов в данной области. Полученные данные были путем тематического анализа распределены по пяти социальным сферам, на которые влияет цифровизация. В результате кластеризации была разработана новая система, которую авторы и соавторы проанализировали на предмет полноты и точности. В ходе двухэтапного процесса достижения консенсуса выявленные детерминанты были распределены по степени срочности принятия политических мер: высокая, умеренная и низкая.

**Результаты:**

Было выявлено 13 804 документа, из которых 204 соответствовали критериям включения. Было установлено, что в ходе цифровой трансформации общества сформировались или изменились 127 детерминантов здоровья (37 цифровых, 33 социальных, 33 торгово-экономических и 24 политических детерминантов). Из этого числа 30 детерминантов (23,6%) были признаны особенно актуальными для принятия политических мер.

**Вывод:**

В этом обзоре представлен всеобъемлющий анализ факторов, определяющих здоровье в цифровой, социальной, торгово-экономической и политической сферах, с особым акцентом на то, как политические решения, индивидуальное поведение и более широкие факторы влияют на здоровье в условиях цифровизации. Полученные результаты способствуют более глубокому пониманию того, как проявляются последствия для здоровья в цифровой экосистеме, и служат основой для разработки стратегий, направленных на решение сложных и развивающихся проблем, связанных с детерминантами здоровья.

## Introduction

Recognizing the social determinants of health, such as age, education, employment, geographical location and housing, has formally linked these determinants to individuals' health status. This recognition has helped to establish health not merely as the absence of disease but as a reflection of everyday living conditions.^1^^–^^4^ Complementary frameworks have been developed for specific domains of health determinants (Box 1), acknowledging that these determinants are essential for maintaining and improving individual and population health. Over time, these determinants have been progressively incorporated into policy-making and governance in countries worldwide.^11^ However, a recent review of health determinants frameworks highlighted the need for a new framework that preserves the core components of existing models while addressing newly emerging health challenges. This new framework should also incorporate recent insights, including those related to digital transformations, which were previously considered irrelevant.^12^

Box 1 **Overview of definitions pertaining to the various domains of health determinants**

***Social determinants of health***

Originally referred to the social, cultural, political, economic, commercial and environmental factors that model the conditions in which people are born, grow, work, live and age, as well as the broader set of forces and systems that affect the conditions of daily life.^3^^,^^4^ In the context of this article, the political and commercial determinants are considered separately due to the availability of specialized literature on these constructs.

***Political determinants of health***

Local, regional, national and transnational norms, policies and practices that emerge from political interactions across all sectors affecting health. These policies and practices can comprise all rules that inform or dictate behaviour, ranging from broad social norms to individual policies (e.g. trade agreements) and practices (e.g. unregulated activities of transnational corporations).^5^

***Commercial and economic determinants of health***

Systems, practices and pathways through which commercial actors influence health and equity. These determinants capture the complex and often negative links between the commercial sector and health.^6^^,^^7^

***Digital determinants of health***

Any factor rooted in, contingent on or inextricably linked to the digital world that can directly or indirectly influence health or well-being. These determinants can change how health care is delivered to improve health, modify existing relationships between social, political or commercial determinants and health, or create entirely new ways to influence individual or population health.^2^^,^^8^^–^^10^

While the impact of digitalization on health and social sectors have been studied extensively, research focusing on how the digital world itself interacts with individual and population health is still emerging.^2^^,^^8^^,^^9^^,^^13^^,^^14^ The World Health Organization's (WHO) *Global strategy on digital health* underscores the need to ground digital foundations within national strategies, establish national digital health agendas and strategies for the health sector, and work with different sectors and stakeholders at all levels of governance – from policy to service delivery to individual decision-making.^15^ In doing so, the global strategy highlights the importance of understanding the state of health determinants during a period of rapid digital advancements and proliferation in society.^16^ The aim of this study was therefore to map what health determinants have manifested in this period.^8^^,^^9^^,^^17^ We also wanted to investigate how existing social, political, and commercial and economic determinants of health are redefined in a digital context, as well as to reach expert consensus on what digital and digitalized determinants of health should be urgently considered at various governance levels. 

## Methods

### Scoping review

We conducted a scoping review to identify articles that outlined how established social, political or commercial and economic determinants of health have changed during rapid digital advancements, or that described emergence of new digital determinants of health. We followed the scoping review framework developed by Arksey & O’Malley and Levac et al.,^18^^,^^19^ and the manual for evidence synthesis for scoping reviews^20^^,^^21^ from JBI. We report our findings according to the PRISMA-ScR guidelines.^22^

#### Eligibility criteria

Eligible articles had to either discuss how social, commercial or political determinants of health changed due to digital transformations, or highlight any new determinants that manifested because of digital transformations. We did not consider articles discussing enablers or barriers of digital health implementation as these were mapped in previous research,^23^ unless they contained information on how certain population groups may experience difficulties engaging with the digital world or how those difficulties might affect individual health. We only considered publications in English. For feasibility purposes, the systematic search was limited to only include systematic, scoping, integrative and realist reviews, as MEDLINE and Embase (192 710 records) and Web of Science (137 785 records) otherwise returned an unfeasible number of records to screen. Furthermore, we only considered publications from 2018 onwards, as this period marks a substantial acceleration of digital transformations, largely driven by the coronavirus disease 2019 (COVID-19) pandemic, rendering the pre-2018 societal landscape less comparable to the current landscape.^9^^,^^24^^–^^26^

#### Search strategy and data collection

We assumed that most articles discussing health determinants are published in health-specific or interdisciplinary journals. We therefore chose to systematically search MEDLINE, Embase and Web of Science, as these databases cover both health-specific and interdisciplinary academic fields.^27^

We synthesized keywords related to the social,^28^^,^^29^ commercial and economic^7^ and political determinants^30^ of health from systematic literature reviews. While no previous research was available to inform our search string for digital determinants of health, previous studies on digital health informed our digital-focused keywords.^23^ We supplemented our systematic search with non-systematic searches using PubMed® and the first 300 hits in Google Scholar.^31^ These additional searches ensured the inclusion of the most recent academic and grey literature on digital transformations in social, commercial, and political determinants, which may not have been included in the identified reviews. An information specialist validated the search strategies, which are available in Box 2. Two authors calibrated the eligibility criteria on a random selection of approximately 10% of the total identified articles before they screened identified records. A third author resolved any disagreements between the two authors. The initial search was performed 2 August 2023 and updated on 24 September 2023.

Box 2 **Search strings used to identify determinants of health that have change or emerged during digitalization of society**

**Systematic searches**

**Medline or Embase**

1. (online or digital or virtual or Internet or AI or “artificial intelligence” or telehealth or telemedicine or ehealth or e-health).ti,ab.
2. Telemedicine/
3. 1 or 2
4. (“social determinant*” or “structural determinant*” or “socioeconomic factor*” or education or income or poverty or employment or housing or gender or ethnicity or race).ti,ab.
5. Employment/ or Housing/ or Poverty/ or Income/ or Education/ or Schools/ or Literacy/ or Socioeconomic Factors/
6. (“commercial determinant*” or ((commercial or corporate) and determinant* and (health or disease*)) or CDoH).ti,ab.
7. “political determinant*.”ti,ab.
8. (democracy or autocracy or “welfare regime” or “welfare state” or “welfare capitalism” or politics or “political tradition” or internationality or globalization).ti,ab.
9. (health or “health service*”).ti,ab.
10. 8 and 9
11. 4 or 5 or 6 or 7 or 10
12. 3 and 11
13. (“systematic review” or “scoping review” or “realist review” or “integrative review” or “umbrella review”).ti,ab.
14. 12 and 13
15. limit 14 to yr = ”2018 -Current”
16. limit 15 to “remove preprint records”

**Web of Science**

TS = (online or digital or virtual or Internet or AI or “artificial intelligence” or telehealth or telemedicine or ehealth or e-health) AND (TS = (“social determinant*” or “structural determinant*” or “socioeconomic factor*” or education or income or poverty or employment or housing or gender or ethnicity or race) OR TS = (“commercial determinant*” or “corporate determinant*” or “political determinant*”)) AND TS = (“systematic review” or “scoping review” or “realist review” or “integrative review” or “umbrella review”)

**Non-systematic searches**

**PubMed®**

(digital[Title/Abstract] OR online[Title/Abstract] OR virtual[Title/Abstract] OR internet[Title/Abstract] OR telehealth[Title/Abstract] OR ehealth[Title/Abstract] OR AI[Title/Abstract] OR “Artificial Intelligence”[Title/Abstract]) AND (“social determinants of health”[Title/Abstract] OR SDoH[Title/Abstract] OR “commercial determinants of health”[Title/Abstract] OR CDoH[Title/Abstract] OR “political determinants of health”[Title/Abstract] OR PDoH[Title/Abstract])

**Google Scholar**

Search 1: “AI” “social determinants of health”

Search 2: “digital” “social determinants of health”

Search 3: “AI” “commercial determinants of health”

Search 4: “digital” “commercial determinants of health”

Search 5: “AI” “political determinants of health”

Search 6: “digital” “political determinants of health”

Search 7: “digital determinants of health”

### Data synthesis and analysis

#### Thematic analysis

We used a thematic analysis to extract data relevant to how social, commercial and economic, or political determinants of health changed or how new digital determinants of health manifested in the context of the digital world.^32^ We continued the data extraction of eligible articles until thematic saturation was reached.^33^ Two authors extracted information on these determinants and clustered them post-hoc into five parts of society affected by digitalization using the 1991 model for social determinants of health as blueprint,^4^ during which we also developed the initial draft of the conceptual framework. During this process, three authors iteratively identified subject matter experts through existing research collaborations, who they invited to join as author or contributors depending on the invitees’ preference.

All authors and contributors reviewed and enriched the findings of the literature review by identifying additional records between 3 October 2023 and 22 December 2023. They also reviewed and refined the conceptual framework during this time to ensure the framework was comprehensive and accurately captured how the period of rapid digital advancements manifested new and redefined existing health determinants across various parts of society. All contributors are listed in the below acknowledgements section.

#### Consensus process

To prioritize the identified determinants for policy action, a two-round internal consensus process was conducted between 8 January and 23 May 2024 among the authors and contributors. To rate the urgency for policy action of the identified health determinants, participants logged in to Welphi (Welphi, Lisbon, Portugal), a web application designed for consensus processes. During the first round (lasting 6 weeks), they were instructed to evaluate each health determinant using a 5-point Likert scale (1: not urgent; 2: somewhat urgent; 3: fairly urgent; 4: urgent; and 5: very urgent) based on the following statement: “Under the recognition that all health determinants are important to address, how urgent is it for this health determinant to be taken into account?” The determinants were presented by societal category outlined in the conceptual framework, and the Welphi application randomized the order of determinants in each category to reduce the potential effect of order bias and scoring fatigue.^34^ To reduce the burden of the consensus process, the second round (lasting 4 weeks), involved only authors and contributors who fully completed the first round, and they rated only determinants deemed of moderate urgency in the first round. After the consensus process concluded, all authors and contributors were invited to validate the description and triangulation of the results.

In the consensus analysis, we included both complete and incomplete responses. We calculated outcome measures as percentages and median values along with interquartile ranges (IQRs) to indicate agreement on the five-point scale. We combined the proportions of 4 and 5 ratings into a urgency percentage.^35^ By combining these measures, we classified determinants into three categories: (i) high urgency, defined by a median rating of 4 or 5, an urgency percentage of 80% or higher and an IQR of 1 or lower; (ii) moderate urgency with a median rating of 4 or less and an urgency percentage of 50–79%, regardless of IQR; and (iii) low urgency, defined by a median rating less than 4, an urgency percentage of less than 50%, and an IQR of 1 or higher. We did the analysis in R version 4.2.3 (R Foundation, Vienna, Austria).

## Results

**Fig. 1:**
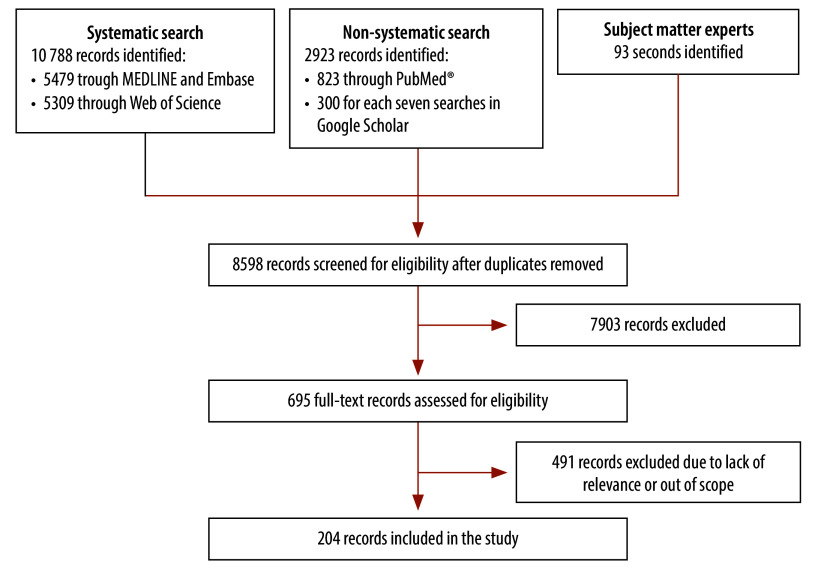
Flowchart outlining the selection of article on determinants of health in the digital age

Our systematic search yielded 10 788 records and our non-systematic searches yielded 2923 records. Subject matter experts identified an additional 93 records. After deduplication, we screened 8598 records for eligibility and included 204 records (Fig. 1). We excluded seven articles during full-text screening, because they were not written in English (listed in the online repository);^36^ these will be screened in future work. The most common reason for exclusion was a lack of relevance in which the record did not address either the impact of digital transformations on health, or how the digital world affected existing structures of health determinants. The crude interrater agreement score between the two reviewers was 93.7% (774/826) and the interrater agreement was moderate (Cohen’s *κ*: 0.663).

### Conceptual framework 

In total, we identified 127 health determinants that manifested or changed during the period of rapid digital advancements and proliferation in society (37 digital, 33 social, 33 commercial and economic and 24 political). We clustered these health determinants into five societal categories affected by digitalization. The first category is person-specific determinants, which includes personal views, perceptions, resources, behaviours and characteristics. The second is community determinants, comprising localized determinants that affect health within a village, city or other local community. The third category is technology-related determinants, which encompass determinants related to digital devices, software and other technologies. The fourth is policy determinants, reflecting policies in specific areas such as health care, transport, education and employment. The fifth is political, economic, societal and cultural determinants, which represent a broader socioeconomic and political climate, including the cultural settings across one or more countries (Table 1; available from: https://www.who.int/publications/journals/bulletin). 

**Table 1:** Overview and glossary of new and updated definitions of health determinants in a digital age

Health determinant	Definition and relevance to the digital world
**Digital domain**	
*Person-specific determinants*	
Device and software availability^13^^,^^14^^,^^23^^,^^37^	The availability and ownership of the necessary hardware required to access digital solutions, such as mobile applications, or to access websites. The complexity of devices can affect how and to what extent different combinations of digital, social, commercial and political determinants can materialize. For instance, mobile phones, smartphones, tablets, wearables, computers and cloud-based services vary in their functionalities, offering different ways to access the digital world
Internet access and connectivity^13^^,^^14^^,^^23^^,^^37^^–^^41^	Whether individual users have reliable access to high-quality internet, which will be required for many digital solutions to function or interact with other software
Problematic device and internet use^13^^,^^42^^–^^45^	The amount of time spent online can lead to adverse health outcomes. Problematic use of internet, gaming addiction, problematic online pornography use, cyberbullying, violence and normative body shaming are examples of how the digital world can adversely affect individual health. Furthermore, increased screen time for children aged 1 year has been associated with developmental delays in communication and problem-solving at ages 2 and 4 years
Digital self-efficacy, empowerment, and altruism^14^^,^^38^^,^^46^	The perception that individual and collective problems can be solved through effective and effortless sharing and use of data and digital solutions, also known as digital confidence
Digital literacy^13^^,^^23^^,^^37^^–^^39^^,^^47^^–^^50^	The ability of individual users to find, evaluate and communicate information produced by digital solutions
Use of virtual private networks^51^^,^^52^	The ability of digital solutions to operate within virtual private networks providing platforms to access pseudo-anonymized data in a secure environment
Cyberbullying^42^^,^^43^^,^^53^	Cyberbullying has negative health effects, which are compounded by the anonymity afforded to propagators. This anonymity often leads propagators to forget that another physical person is on the receiving end of the harassment, which can magnify the volume and intensity of the abuse. Unlike traditional bullying, victims of cyberbullying have no safe space to escape, as the abuse only ends when the aggressor chooses to stop. In cyberbullying, the abuse can theoretically be constant, all hours of the day, especially when large audiences are involved, leading to exponential spread of abusive messages
Attitude towards digital solutions^23^^,^^24^^,^^38^^,^^39^^,^^54^^,^^55^	The willingness to engage with digital solutions, which can vary between different population groups and depends on the intended purpose and usability of the solutions. For example, younger populations may be more willing to engage with digital solutions than elderly populations
*Community determinants*	
Provision of digital training ^14^^,^^23^^,^^56^^,^^57^	Digital training courses can improve digital skills regarding navigating the digital world, safe digital and data practices and behaviours. These courses can be offered to all users and prospective users of digital solutions, including citizens, patients, and health workers. Digital training courses have been shown to increase knowledge and performance of health workers and increase efficiency in the delivery of skills upgrades
Data and digital capacity^9^^,^^58^	Institutions and organizations should build an infrastructure to securely store, analyse and act on the data they collect, while ensuring the privacy and safety of data sources, such as citizens or patients. Moreover, it is imperative to have adequate human resources and capacity to design, develop, implement and sustain digital and data solutions. The workforce must have sufficient technical expertise to conduct this work
Digital penetration and implementation incentives^23^^,^^59^^,^^60^	The extent to which digital solutions are adopted within a social or organizational setting can be facilitated with financial and non-financial incentives. Financial incentives, such as dedicated funding compensation to offset high fixed costs, can partially remove the financial barriers to embracing digital transformations
Infosphere^8^^,^^9^^,^^42^^,^^47^^,^^61^^–^^68^	The type and extent of information available within a given environment can affect health-related decision-making. For example, research on antivaccine movements has shown that the spread of mis- and disinformation can have real-world health effects. A similar effect has been observed with anti-immigration sentiments shared and promoted online, which negatively affect migrant communities. Conversely, the public health sector can leverage social media and digital marketing to promote health and disease prevention campaigns, as shown during the COVID-19 pandemic when WHO and national governments used social media to distribute health and safety messages. However, disinformation on social media negatively influences vaccination coverage and increases the likelihood of negative discussions on vaccines. Social media both expose individuals to inaccurate health information and harmful content, while offering a platform for public, patient and health professional interaction, altering the nature and speed of health-care communication. Hyperconnectivity through social media may displace in-person relationships and healthy behaviours such as physical exercise. Non-factual and misleading information about COVID-19 vaccination and pervasive anti-vaccine content continue to proliferate on social media platforms. Social media may also exploit pre-existing behavioural patterns, encouraging individuals to spend considerable time online. As access to digital technology and content delivery channels increases, individuals are exposed to more information. This exposure might lead to information fatigue, even when content is high-quality, which may reduce individuals’ attentiveness to any messages they receive, even important health-related messages
Implicit technology bias^38^	The impact of unconscious perceptions held by digital developers and health workers of an individual’s digital literacy, technology access, attitudes towards use and willingness to engage with digital tools
*Technology-related determinants*	
Gamification^42^^,^^43^^,^^69^^–^^72^	Videogames often incorporate gambling elements that can jeopardize the health and financial stability of players. In contrast, videogames are also being deployed to address stress and anxiety or aid in educational delivery
Moderation of harmful content and misinformation^8^^,^^42^^,^^73^	In the digital world, people may be exposed to risky content, such as gambling, violence, social media–based bullying, terrorist and violent extremists’ content and normative body shaming. Similarly, people tasked with moderation might have poor mental well-being due to prolonged exposure to specific content and limited workplace support
Explainability^9^^,^^74^^–^^78^	AI has introduced a shift towards probability-based medicine that is based on statistical interpretation of data, which can blur transparency and accountability in medicine and public health. Explainability refers to the characteristic of an AI-driven system allowing a person to explain and to reconstruct the predictions presented by AI technologies
Ambient intelligence^14^^,^^38^	Digital tools and software can benefit the monitoring and management of chronic conditions that require frequent or constant monitoring (for example, hypertension, diabetes, congestive heart failure or chronic obstructive pulmonary disease) by improving or enhancing monitoring hardware through digital solutions. Digital tools can also simplify interactions with health and social services, for example, through online appointment bookings and consultations or recurring electronic prescriptions
Model accuracy and algorithmic validation^14^^,^^38^^,^^75^^,^^79^^–^^82^	Reliability of outcomes and information produced by AI models. The representativeness of the data used to train the AI model will influence its reliability for the population it is intended to serve. The biases embedded in the data set that an algorithm is based on (algorithmic bias) can skew how AI affects health and how this effect can differentiate across population groups. Algorithms trained on biased data may be less effective, either by being overly sensitive to certain population groups or failing to detect diseases that manifest differently across population groups
Personal customizability^23^^,^^55^^,^^83^	Digital tools need to be adjustable to personal needs and preferences to mitigate the risk of excluding individuals from the digital world. For instance, people with sensory or cognitive impairment might require specially adapted digital interfaces, and those with poor literacy might need options that are visually easy to interpret and use
Data and digital interoperability^9^^,^^23^^,^^54^	The ability of digital tools to communicate and exchange information with other information systems and software, such as electronic health records or other digital solutions inside and outside of the health-care domain. This ability can be operationalized using common data standards (for example, ICD, SNOMED-CT, ISO, NUTS and OMOP). Similarly, this determinant captures how digital solutions or services can fit within a broader digital or organizational ecosystem
Reliance on internet^23^^,^^40^	Digital solutions that can function for a set amount of time without being connected to the internet can be more suitable in settings with limited internet access
Security settings and features^84^	Functionality of digital solutions which protect patient data, and prevent inappropriate sharing of data with third parties including for commercial or fraudulent purposes. These features can also function to protect vulnerable groups from harmful digital exposure, such as parental controls protecting children using digital apps and devices
Firewall protection^85^	A firewall, which can be hardware, software or both, is typically used for monitoring the network traffic to allow or block the traffic using a set of rules. In commonly used packet-filtering firewalls, policy rules are implemented to monitor changes to the network and preserve the required security level. However, with the rapid increase of devices and the corresponding increase in policy rules, firewall policy anomalies occur more frequently, putting user data at risk
*Policy determinants*	
AI validation, transparency, explainability, accountability and ethics^9^^,^^74^^–^^76^^,^^79^^,^^80^^,^^86^	Health workers frequently adopted an outcomes-based approach to the ethical integration of AI technologies in practice. That is, if these technologies do more good than harm, they are considered ethical. However, modern medicine is built on transparency in decision-making, which AI could obscure due to its black-box characteristics, potentially violating medical ethics and undermining patient trust
Data consent policy^8^^,^^87^^,^^88^	The use of appropriate consent protocols for the collection of data in commercial (for example, data tracking in online advertising), health care or research-focused digital solutions. It is important to consider how consent applies when data from one context is used within another, such as using commercial or health-care data in research. In health care, these protocols should ensure that consent is asked where appropriate, without interfering with the delivery of high-quality care
Privacy and security policy^13^^,^^74^^,^^75^^,^^86^^,^^89^^–^^93^	The extent to which an individual's data are protected from (cyber)security threats or inappropriate sharing of confidential data either by accident or for commercial purposes. Privacy and security requirements and protocols for personal health data should be in accordance with legislative or institutional policies, as well as best practices. In AI, it raises the question of how AI can be trained using sensitive data, ensuring privacy and maintaining the confidentiality of the patients or citizens whose data are used for training purposes
Access and sharing policy^13^^,^^74^^,^^75^^,^^86^^,^^89^^–^^93^	The extent to which individuals are empowered by policy to control the data they share and determine which parties have access to that data. Access policy should include guidance for data controllers on measures like anonymization or pseudonymization of electronic data when using health-care or commercial data for secondary purposes (for example, research, public health surveillance and monitoring), as well as the appropriate use of privacy-enhancing technologies
Mis- and/or disinformation policy^9^	The extent to which legislation is implemented that aims to address, mitigate or eliminate the spread of mis- and/ or disinformation in traditional and online media platforms
Outcomes, utility and value sharing^23^^,^^58^^,^^94^^,^^95^	Digital solutions will be able to trace data utility back to the providing sources and determine how the created value would be best shared for public interest. Given the volume and personal nature of data, this ability of tracing poses a unique consideration for the health-care field
*Political, economic, societal and cultural determinants*	
Public–private–person partnerships^54^^,^^96^^,^^97^	The extent to which public–private partnerships are present in the societal environment and how the perspectives of individual citizens are included as part of these partnerships, as well as the extent these partnerships are encouraged or obstructed in the context of digital transformations, including within the health-care system. Public–private partnerships have the potential to develop novel health technologies, by combining data on population health needs generated in the public sector with the innovation and manufacturing capabilities of the private sector. Digital technology applications offer opportunities to evolve the relationship between the private and public sectors in recognition that both are needed to resolve larger challenges
Digital divides^8^^–^^10^^,^^17^^,^^54^^,^^55^^,^^83^^,^^98^^–^^100^	Discrepancies between population groups, regions or countries in access to internet and digital devices; gaps in use due to different levels of digital literacy and skills; and differences in health outcomes between population groups resulting from the use of digital technologies
Financial investments and conditions^14^^,^^23^^,^^54^^,^^59^^,^^89^^,^^101^	The extent to which and conditions whereupon financial investments are made into digital transformations and what sectors and societal problems these investments focus on. For example, to what extent financial investments are targeted towards addressing social determinants of health, prevention, and health promotion versus treatment options that have a high return on investment, for example oncology or immunology
Data governance and ethics^8^^,^^9^^,^^14^^,^^38^^,^^79^^,^^89^^,^^92^^,^^102^^–^^106^	The way data are operationalized in society. This determinant encompasses a broad range of concepts, such as how individual data are managed and stored; the extent to which people retain data autonomy; the rights of data subjects and data controllers; how pooled information from raw data can be made accessible to drive scientific innovation and public health monitoring and surveillance without compromising data security; the extent to which data is used to generate public value or private profits; and how big data and sophisticated analytics can be deployed to address societal challenges
Data culture^9^	A culture of collecting, collating and analysing large volumes of data to predict outcomes in the health care, social, economic or political contexts such as disease prediction, stock market prediction or prediction of election results
Digital public infrastructure^14^^,^^17^^,^^23^^,^^55^^,^^65^^,^^100^^,^^107^	The extent to which the digital public infrastructure, such as broadband, mobile phone reception and hardware, reaches the entire society without the risk of exclusion. Additionally, the extent to which data are being housed in siloes. A system-wide approach to application and architecture design that prioritizes the development of an integrated and interoperable framework is generally more effective than a piecemeal approach, which can lead to fragmented and isolated digital tools
Right to scientific advancement^9^^,^^108^	The International Covenant on Economic, Social and Cultural Rights is binding and customary international law, which states in Article 15 that all people have the right to enjoy the benefits of scientific progress (Article 15(1)(b)) and the right to have the freedom to participate in scientific advancement and innovation (Article 15(3)). The extent to which individuals and policy-makers are aware of and operationalize this right can influence population health. This right is a positive obligation, meaning states are required under international law to take steps to ensure its realization
Regulatory mandate^23^^,^^100^^,^^109^^,^^110^	The capacity and ability of health ministries to work across ministries for policy-making, standards-setting, planning implementation of digital solutions and supportive infrastructure. However, stewardship for health lies with health ministries; and in some countries competencies to plan, partly regulate and even implement digital health lies with health ministries. This determinant also captures the function of regulatory institutions to monitor the efficacy and safety of the digital world. Importantly, regulations aimed at the digital world must be strict enough to protect patients and citizens, yet agile enough to incentivize further development of digital innovations across sectors. This determinant also includes the ability to create environments where implementers and regulators can safely explore regulatory, implementation and delivery mechanisms of the digital world, allowing them to collaboratively navigate and understand these complexities
**Social domain**	
*Person-specific determinants*	
Health literacy^9^^,^^37^^,^^111^	The ability to obtain, read, understand and use health-care information to make appropriate and informed health decisions is increasingly becoming a core skill for health-related information on the internet. Digital interventions could potentially improve knowledge, attitudes, empathy and decrease stigma regarding people struggling with ill health
Education level^9^^,^^17^^,^^112^	Individuals’ education level is highly correlated with their digital literacy and health literacy
Race and/or ethnicity^13^^,^^23^^,^^59^^,^^113^^,^^114^	Technology and internet use patterns differ by race and/or ethnicity, which can limit ability to maximize benefits from digital health solutions
Housing^13^^,^^40^	Stable housing is essential for consistent access to the digital world, as well as for receiving necessary hardware to access specific digital solutions, such as tablets sent to a person’s home for the duration of their treatment
Migration status^66^^,^^115^^–^^119^	Migrants are affected by social inequalities and often encounter experiences during the migration process that put their physical, mental and social well-being at risk. They often face poverty and social exclusion, which negatively influences their health. Migrants’ health is also largely determined by the availability, accessibility, acceptability and quality of services in the host environment. Digital technologies can be essential for refugees to claim their rights, such as the right of information and expression, the right to cultural identity maintenance, and the right to protection, citizenship and well-being in the host country
Legal identity^120^^–^^122^	Traditionally, the transaction of, for instance, money or data, did not require a person to have a single, formal identity and using multiple names was acceptable unless fraud was involved. However, in the digital era, this scenario has shifted. Transactions that used to be face-to-face, often with a background of personal familiarity, are now conducted remotely through technology. This shift in transaction methods has emphasized the importance of identity, especially digital identity. Furthermore, the lack of a legal identity can be a critical barrier to accessing digital technologies
Health and disability status^49^^,^^55^^,^^59^^,^^112^^,^^123^	People living with disabilities often face greater barriers to accessing the internet, while those experiencing poor physical or mental health, or psychological distress tend to more intensely and frequently use the internet
Employment status^9^^,^^124^^–^^127^	Unemployed populations have lower levels of digital literacy as baseline digital literacy is often required for many jobs. The digital world also gave rise to novel forms of employment (e.g. gig workers or digital platform workers)
Sex^59^^,^^123^^,^^128^	Males tend to be at a higher predisposition of problematic internet use than females. Females can experience more structural barriers to access the digital world depending on their location and social and cultural environment
Science literacy^129^^,^^130^	A scientifically literate population relies on evidence to evaluate the quality of information. In the digital era, science literacy can be understood as including three dimensions that span the lifecycle of science information: (i) civic science literacy, which involves understanding how science is produced and how it relates to broader society; (ii) digital media science literacy, which focuses on how scientific information appears and circulates through media systems; and (iii) cognitive science literacy, which pertains to how people interpret science information they come across
Age^8^^,^^9^^,^^13^^,^^17^^,^^23^^,^^46^^,^^59^^,^^112^^,^^131^^,^^132^	Younger people have better access to the digital world and more sophisticated digital skills
Urbanicity^9^^,^^17^^,^^59^	Urban populations have greater access to health-care services and internet coverage, making them less vulnerable to digital health exclusion, which is more commonly experienced in rural populations
Physical activity^13^^,^^123^	People with sedentary lifestyles tend to be more frequent users of internet
Access to health and social services^54^^,^^100^	Access to sufficient high-quality health-care and social services in one’s vicinity is a key determinant of health. In the digital age, one needs to determine how much these services rely on digital technologies and how they accommodate population groups that are less digitally skilled or willing to use
Food security^132^	Online shopping and online food purchasing have created new options for people to ascertain their food security
Impulsivity^43^^,^^123^	Impulsivity is characterized by failure in inhibiting potentially risky impulses for the individual or their surroundings. Impulsive people have a predisposition for problematic internet use. Conversely, people having a problematic internet use tend to display poorer levels of impulse control
Social skills^43^^,^^123^	Social skills refer to a person’s ability to interact with others in their environment. People with higher social skills tend to exhibit lower levels of problematic internet use, while lonely people are more like to experience it
Emotional regulation^43^	Emotional regulation involves the processes of monitoring, evaluating and modifying emotional reactions. People with poorer emotional regulation skills are more prone to problematic internet use. More hostile adolescents also showed a predisposition for problematic internet use
Gender identity^23^^,^^59^^,^^114^^,^^128^^,^^133^^,^^134^	People with minority gender identities may rely on participating in society online, such as in education and employment, to decrease the risk of discrimination and harassment. Conversely, these population groups are at major risk of cyber-harassment due to their gender identity
*Community attributes*	
Social support^13^^,^^23^^,^^39^^,^^81^	Peer-to-peer support for patients and health workers can improve digital literacy and motivation to maximize use of digital solutions. However, social networks and support systems might be weaker among disadvantaged groups. Social support can also be found through online communities of digitality literate populations
Organizational culture^23^	A supportive organizational culture fostered by strong and committed leadership can influence the uptake of digital solutions within health-care settings
Institutional workflow^23^	The ability to easily integrate digital solutions within pre-existing workflows without requiring large-scale changes can maximize uptake and usage of such solutions
Propensity to change^23^	A risk-averse environment may resist change, hindering the introduction of digital technologies. Conversely, approval from the institutional or social environment can be an important indicator of the uptake and use of digital technologies
Community participation and engagement^23^^,^^135^	Efforts by digital developers and purchasers to facilitate participation of users during implementation can maximize uptake and use of digital solutions. Examples include involving patients and health workers in implementation strategies for electronic health records
*Technology-related determinants*	
Inclusive design^23^^,^^48^^,^^54^^,^^55^^,^^59^^,^^75^^,^^83^^,^^113^^,^^136^^–^^138^	Involvement of a wide range of end-users within the development and implementation of digital solutions can improve the usability of digital interfaces and acceptability among different populations. Digital tools should be co-designed and co-implemented in collaboration with end-users to maximize the likelihood of their uptake by the relevant stakeholders, with the recognition that engagement might differ across population groups in different countries. However, this approach is scarcely used in the context of AI. The approach also ensures that the characteristics of the existing system are considered in the implementation process, and that the implementation is understood by people of multiple skill levels that operate within the organization
Good practice design^23^^,^^65^	The design of digital solutions should conform to sector-specific guidelines and protocols to ensure that the digital solution is based on the best practices of the respective sector, for example, clinical guidelines in health care
*Policy determinants*	
Employment and labour policy^9^^,^^13^^,^^38^^,^^126^^,^^127^^,^^139^	The organization of employment has been slowly shifting over the last two decades with job applications almost exclusively being available online. During COVID-19, many sectors were also forced to migrate to the digital space to remain operational during national restrictions. Novel employment opportunities have also emerged, such as digital platform work, that are dependent on having digital devices to conduct the work. Prospectively, technology adoption will remain a key driver of business transformation. Big data, cloud computing and AI are among the most likely technologies to be adopted, leading to considerable changes in the employment market
Health and social care policy^9^^,^^13^^,^^23^^,^^38^^,^^101^^,^^140^^–^^142^	The prioritization of digitization within national and regional health policy and planning has considerable influences on access to and dissemination of digital health solutions, such as electronic health records and medical devices. Many countries have national digital health strategies to facilitate objectives surrounding the implementation of digital technologies in the health sector. However, a major bottleneck in digital health transformation is the development of reimbursement and financing mechanisms and the delayed inclusion of digital health in insurance policies. When digital health is included in insurance coverage, usage tends to increase. For example, at the start of the COVID-19 pandemic, digital health usage among Medicare recipients in the United States increased from approximately 13 000 virtual visits per week before the public health emergency was declared to nearly 1 700 000 virtual visits in the last week of April 2020.^142^
Education policy^13^^,^^102^^,^^143^^–^^148^	Curricula can include digital skills training that positively affects the digital determinants of health. However, there is a risk that such training may exacerbate digital exclusion if it does not consider individuals with special educational needs. Digital learning facilitates just-in-time learning, puts the student in charge of his/her learning process and enhances time efficiency. Nevertheless, the lack of social interaction with peers and teachers is not easily overcome by using digital communication channels, such as internet forums and email
Urban–rural planning and development^8^^,^^17^^,^^59^^,^^149^^–^^151^	The extent to which greening digital practices are integrated within urban planning. Examples include providing facilities and platforms for recycling of digital health wearables, robotics and devices, as well as implementing regulations to reduce the environmental impact of data centres and servers. When doing so, it is important to ensure that the needs of rural communities are considered and that policies and actions are not limited to urban settings, because rural communities experience more barriers in accessing the digital world
*Political, economic, societal and cultural determinants*	
Cultural and social norms and values^9^^,^^38^^,^^54^^,^^59^	Social norms and values are the set of beliefs and philosophies that affect who develops digital tools, what is developed, how it is used and who it is used by. These development considerations are influenced by whether there is a culture of data justice and equity that provides citizens with confidence that data is used to improve societal well-being
Religion^152^	Certain religious communities foster fear of potential negative consequences of digital solutions and internet use, resulting in limited or restricted access to such solutions and mobile devices
Socioeconomic inequalities^23^^,^^48^^,^^54^^,^^55^^,^^79^^,^^149^	Populations from more deprived groups experience several barriers to accessing digital health solutions including lower levels of digital literacy, reduced internet access and fewer resources to purchase medical devices or to fund subscription costs
**Commercial and economic domain**	
*Person-specific determinants*	
Financial literacy^81^	The knowledge and skills needed to make informed financial decisions and navigate effectively in the financial system. The digital world has provided novel ways for people to interact with the financial market, such as online exchanges and digital currencies
Consumer literacy^153^	Consumers’ ability to perform consumption-related tasks within a speciﬁc market context. Consumers who rank low on digital literacy may not locate, assess and digest the online information necessary for making a decision. Modern consumers interact online, provide consumer feedback and create content regarding their consumption, which also requires a certain level of digital literacy
Financial stability^81^	Achieving a state where financial resources are well-managed and effective budget allocation decision are made. The stability can be jeopardized by direct-to-consumer marketing or nudging towards financially risky behaviours, such as online gambling
Access to financial services^6^^,^^9^^,^^81^	The proliferation of digital financial services, for example mobile banking apps or mobile-only banks, can reshape access for population groups, particularly in regions where traditional financial services are limited or unavailable
Online spending habits^8^^,^^42^	Tendency to invest in digital health technologies from web-based sources, affecting overall individual spending on health care and the availability and distribution of digital health products
*Community determinants*	
Supply chain^6^^,^^154^^,^^155^	The process of manufacturing and distributing goods and its implications on market penetration. Issues with the supply-chain can lead to inconsistent access to digital solutions or medical devices. Similarly, digital transformations can give rise to new opportunities for illicit goods to penetrate the market
Corporate governance^156^	The way corporations organize their internal governance structure, how disclosure practices have changed in the digital era, how shareholders are engaged through digital technologies, and how fundraising practices have evolved through digital technologies
Enterprise architecture^157^^,^^158^	Enterprise architecture is a framework of principles, methods and models used to design and realize an organization’s business process, information systems and digital infrastructure. Often described as a master plan, it provides a holistic view, addressing complexity management through standardization and consolidation. The plan also provides transparency by simplifying the organizational structure and internal interactions
Market coverage of digital strategies^159^	The extent and reach of a company's efforts to capture a specific target audience and address the needs and preferences of customers. Additionally, this coverage refers to the implementation of a digital health strategy across a wider population, as opposed to fragmented adoption within the health-care system
Information and communication technology reliance^39^	The extent to which people are dependent on technology for various aspects of their daily life, for example to shop for essentials, for banking purposes, to complete tasks related to employment or to communicate with their social networks
Labour practices^6^	Harmful and unsupportive work conditions that can negatively affect employees' physical and mental well-being. The rise of remote work in the digital age has resulted to more sedentary habits, increased isolation, but it also increased flexibility in working hours and location, reduced commuting and decreased the carbon footprint
Waste management^6^	Practices to ensure the responsible disposal of electronic equipment and materials to mitigate potential adverse effects of electronic waste on the environment and public health
Change management^158^^,^^160^	Practices to facilitate the transition to a future state where digital technologies are embedded into processes and operations
Scientific practices^6^	Digital developers who manipulate the scientific process to produce favourable outcomes of their digital solutions can negatively affect health outcomes. Conversely, digital developers who adhere to professional standards in research and evaluation can positively affect health outcomes. Additionally, aiming for inclusive and representative trials across population groups ensures that digital solutions are not disproportionately tailored to one subgroup of the population
Reputational management^6^	Digital developers who achieve and maintain high levels of legitimacy and credibility will find it easier to promote uptake and usage of digital solutions among the public and health workers
*Technology-related determinants*	
Product design philosophy^161^	A product design philosophy reflects the values guiding the development of a digital health technology, such as patient-centredness, user-friendliness, effectiveness and safety. The design of social media applications, including its addictive features, is often driven by profit maximization at the expense of public mental health, particularly among young people
Corporate social responsibility^161^	Actions taken by a corporation to make positive contributions to society beyond their economic objectives, often with the intention of enhancing the company's public perception
Online health-harming goods and service retail^8^^,^^42^^,^^45^^,^^155^	The digital era created new possibilities to disseminate products and services that can adversely affect individual and population health. Notable examples includes online sales of alcohol, drugs, counterfeit medicine and largely unchecked availability of pornography
Dark commercial patterns^162^^,^^163^	Business practices employing elements of digital choice architecture that subvert consumer decision-making, for example, pressuring a purchase with a fake countdown timer, have risen especially since the COVID-19 pandemic
Targeted marketing and nudging^6^^,^^8^^,^^42^^,^^43^^,^^163^^–^^166^	The ability of manufacturers to directly sell to consumers affects health outcomes and health equity. Moreover, it raises ethical dilemmas, including concerns about data security and information accuracy. Marketing practices can drive demand for products and practices harmful to health while also exaggerating structural inequalities by targeting specific geographical areas and vulnerable population subgroups
*Policy determinants*	
Service provision^167^	The provision of the technology, along with necessary support, maintenance and ongoing assistance to ensure successful adoption and use of the digital health solutions
Intellectual property and patent policy^161^	Protection granting creators exclusive rights to use and distribute their inventions. This protection has implications on innovation, market coverage and availability of new products
Regulation of market and marketing strategies for digital contexts^164^^,^^167^	Policy action targeting industries can help curbing marketing efforts by private companies, which may adversely affect health if left unchecked
Economic and financial policies^6^	Policy action, such as regulating advertising and enforcing legal age limits, can help reduce or remove addictive and harmful content that exploits on commercial instincts, such as online gambling
*Political, economic, societal and cultural determinants*	
News and media^164^^,^^165^	Influence of news and (social) media on health is twofold: (i) public health initiatives can use digital channels to share health education, implement behavioural interventions, monitor disease outbreaks and expand the reach of public health efforts; and (ii) the nature of social media platforms creates a conflict of interest between profit and public health. Social media is supported by advertisements seeking to modify behaviour, sometimes promoting harmful practices
Environment^102^	The growing use of digital health solutions has considerable negative impacts on long-term environmental sustainability. The production and disposal of wearable technologies, robotics and devices can cause environmental degradation. Large servers storing data and telehealth communication centres require substantial energy. Negative environmental impacts can ultimately affect health through lack of access to clean spaces and increasing climate change
Commercial influence^6^^,^^168^	Commercial influence refers to the power of business over public opinion, market dynamics and consumer behaviour, which has increased considerably through the ability of businesses to interact directly with a wide audience through digital channels
Degree of privatization^6^^,^^169^	The extent to which the provision or financing of digital solutions and the systems in which they operate are perceived as a public good versus a product created for maximizing profits by private corporations
Financialization^6^	Allocation of financial resources and investment into the development of digital health technologies with the expectation of a return on investment
Lobbying^159^	The process of influencing choices in policy-making by shaping preferences or inducing uncertainty regarding potentially harmful actions. Lobbying can influence how digital solutions are perceived by society, regulated, reimbursed and implemented
Economic stability^81^	Economic stability or instability affects investment, spending budgets and allocation for funds on digital health technologies, which directly influence market penetration of these products
Internationalisation of trade and investment^159^^,^^170^	The level of internationalization in the trade of digital goods, leading to increased demand and regulations, such as trade agreements, tariffs, quotas, supply chains and market access, influences the global dissemination of various digital solutions
Political economy of globalization^159^	Degree to which economic activities and policies are influenced by and in turn influence political decisions and power dynamics on a global scale, including foreign investment, international finance, labour migration, cultural exchange and the role of multinational corporations. The political economy can also influence the global dissemination of different digital solutions
**Political domain**	
*Person-specific determinants*	
Civic literacy^39^	The degree to which an individual possesses the skills and knowledge to participate in public deliberation through the digital world
Epistemic competence^102^	The extent to which an individual can critically evaluate, suggest solutions and effectively communicate regarding public policy changes and development, including the field of digital transformations. This competence also influences their sensitivity to false equivalences and political or commercial pressure
*Community determinants*	
Political messaging^9^^,^^165^^,^^171^	The communication efforts of political parties or organizations in getting their messages across or framing issues to gain trust or alignment with a particular stance
Ownership of technology^2^^,^^9^^,^^149^^,^^169^	The extent to which ownership of data or technology is held by private business as opposed to governed by the state, and its implications on prevailing ideas, priorities and initiatives in global public health
Political engagement and agenda setting^172^	The extent to which the technology enhances political engagement and increase of civil society’s involvement in decision-making processes. Similarly, the extent to which patient communities or networks are engaged in advocating for the development of digital aspects of society
Regional networks^15^^,^^157^	Regional networks are ensconced in the WHO *Global Strategy for Digital Health 2020–2025* and positioned between global frameworks and country initiatives. These networks serve as platforms for knowledge exchange and resource sharing, allowing countries, often working in isolation, to collaborate and agree on common frameworks, such as data interoperability for COVID-19 or digital certification for security
Enfranchisement of marginalized groups^9^^,^^23^^,^^54^^,^^173^	The extent to which historically excluded groups, such as young people, people living with disability or chronic conditions, people from minority gender, sexual or ethnic groups, are integrated in the political decision-making process. They must have the opportunity to participate in decisions that affect their futures, including the design and governance of digital approaches and other data-driven services
Power asymmetries^102^	The unequal distribution of power among decision-making bodies, both directly and indirectly related to public health. In digital transformations, this power largely lies in controlling the public narrative, which has largely focused on the positive potential of digital transformations, while the associated risks remain underdiscussed
*Technology-related determinants*	
Political biases^165^	The degree to which political controversial stances such as gender-affirming care, abortion, contraceptives and vaccination, are incorporated or omitted from digital health solutions
Extension of public space^2^	Digital platforms provide spaces for people across the world to connect, deliberate and support each other, exchange thoughts and ideas, and carry out policy dialogues
*Policy determinants*	
Governance of commercial markets^6^^,^^9^^,^^174^^,^^175^	Commercial markets in different sectors cannot be addressed using a single set of policies. As such, sector-specific policies are vital to ensure that digital markets in various sectors do not, or minimally, adversely affect individual health
Defence, security and justice^176^^,^^177^	Cybersecurity threats are becoming increasingly common and costly, making robust cybersecurity policies and strategies essential for providing a high-level protective layer for the health and safety of citizens in the digital world. Similarly, policy should enable the use of data and digital technologies to benefit individuals, communities and societies
Political influence on campaigns^178^	The degree to which electoral considerations influence the agenda-setting and implementation of digital health policies
Commodification and product-focus of science and technology^102^	By overemphasizing the need for science and technology to yield products and commodities, political power can become concentrated, leading to a neglect of the normative, structural, systemic and historical dimensions of governance. This product-focused framing of technology and innovation can, for example, promote a paradigm that advocates for the infinite growth of digital transformations, an unstainable approach in a world with finite resources
*Political, economic, societal and cultural determinants*	
Digitalization agenda^9^^,^^13^^,^^23^^,^^55^	A political system's belief in the role of digital health technology to improve health outcomes and optimize resources utilization within a health-care system
Geopolitical landscape, competition and collaboration^149^^,^^159^^,^^171^	The potential shift of global power dynamics towards entities with data control or digital technology market dominance might influence implementation and impact of new technologies. For example, implementation of digital technologies in Africa funded by Meta are equally gatekept by Meta
Scientific autonomy and independence^179^	The level to which institutions retain their scientific autonomy while avoiding political and commercial influences. This idea is closely linked to the inherent uncertainties of science and the public's ability to understand these concepts. Scientific independence has been threatened by partisan political interference aimed at gaining voter confidence, underscoring the importance of education to promote societal understanding of science and scientific principles
Accountability and transparency of commercial interests^2^	The policies in place aimed at defining accountability and transparency requirements for the (digital) actions of parties with commercial interests
Local political environment^110^^,^^161^	Coalitions between local and national governments, relevant ministries, organizations, health equity groups and other political actors, along with their lobbying efforts, voter engagement and turnout, can exert pressure on decision-makers to prioritize digital transformation items on the political agenda. Conversely, depending on their stakeholder position, these groups can also generate pressure to keep digital transformation items off the political agenda. This determinant also captures the ability of local governments to achieve their objectives given the available capacity and resources
Open and transparent decision-making^9^	Civic-oriented digital technologies, such as online dialogues and citizen consultations or open government data, can improve public participation in democratic and decision-making processes. These technologies are increasingly seen as enablers of improved public policy and service delivery
Political origins of existing inequalities^159^	The extent to which political institutions or actions, particularly in budget allocation and prioritization, have led to unequal distribution of digital health resources. Decisions about infrastructure, funding and health policy have a major impact on health outcomes
Corporate capture^167^	The extent to which political decisions are influenced by the interests of for-profit business through lobbying, marketing or trade agreements can negatively impact health. This bias happens when policy-makers prioritize digital developers with promising financial prospects over those focused on population health and societal well-being
Development of digital government^9^	Extent to which digital technologies are leveraged in the operations and management of governmental procedures
Sensitivity to false equivalence^102^	The extent to which decision-makers can discern the varying levels, degrees and trustworthiness of evidence when drafting policies on digital technologies. They must be aware of false equivalences, a flawed reasoning where equal weight is given to arguments backed by concrete evidence, and those that are conjecture, untrue or unjust

AI: artificial intelligence; COVID-19: coronavirus disease 2019; ICD: International Statistical Classification of Diseases and Related Health Problems; ISO: International Organization for Standardization; NUTS: *Nomenclature des Unités territoriales statistiques*; OMOP: Observational Medical Outcomes Partnership; SNOMED-CT: Systematized Medical Nomenclature for Medicine–Clinical Terminology; WHO: World Health Organization.

**Fig. 2:**
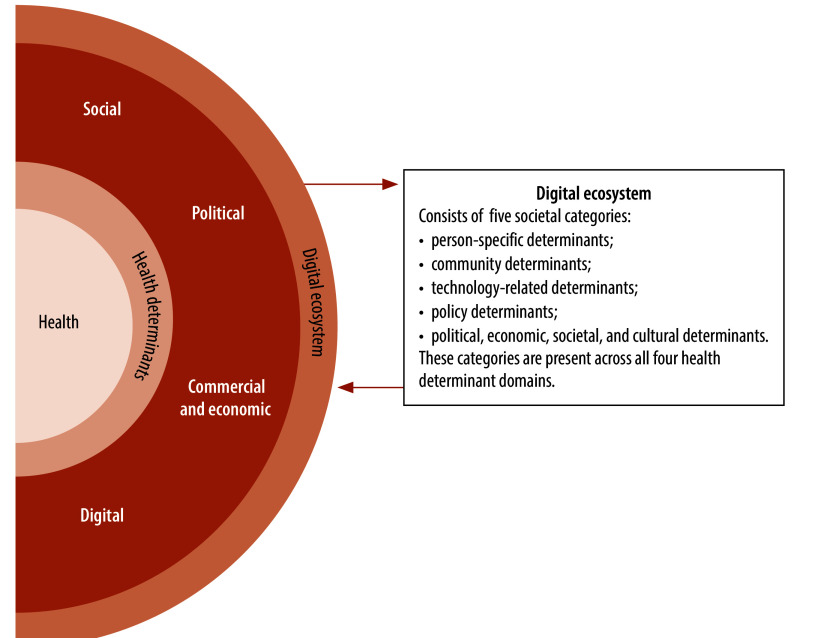
Conceptual framework on the digital determinants of health

The conceptual framework resulting from this categorization is presented in Fig. 2. The framework illustrates the relationship between health and social, commercial and economic, political and digital determinants, as well as how these determinants operate in different parts of a digital ecosystem. With health at the core, the first layer emphasizes that health determinants form a blended, interconnected spectrum that can affect health directly and indirectly.^9^^,^^13^^,^^23^ The second layer classifies individual determinants into social, commercial and economic, political and digital determinants of health.^3^^,^^7^^,^^180^ The outer layer illustrates that these determinants now exist within a digital ecosystem, meaning they interact with individuals through both the physical and digital world. This layer also highlights the disruptive and transformative effects of digital transformation on the social, commercial and economic and political determinants of health that predate the period of rapid digital advancements and proliferation in society,^2^^,^^9^ while simultaneously manifesting a completely new domain in the digital determinants of health.

### Key health determinants

**Fig. 3:**
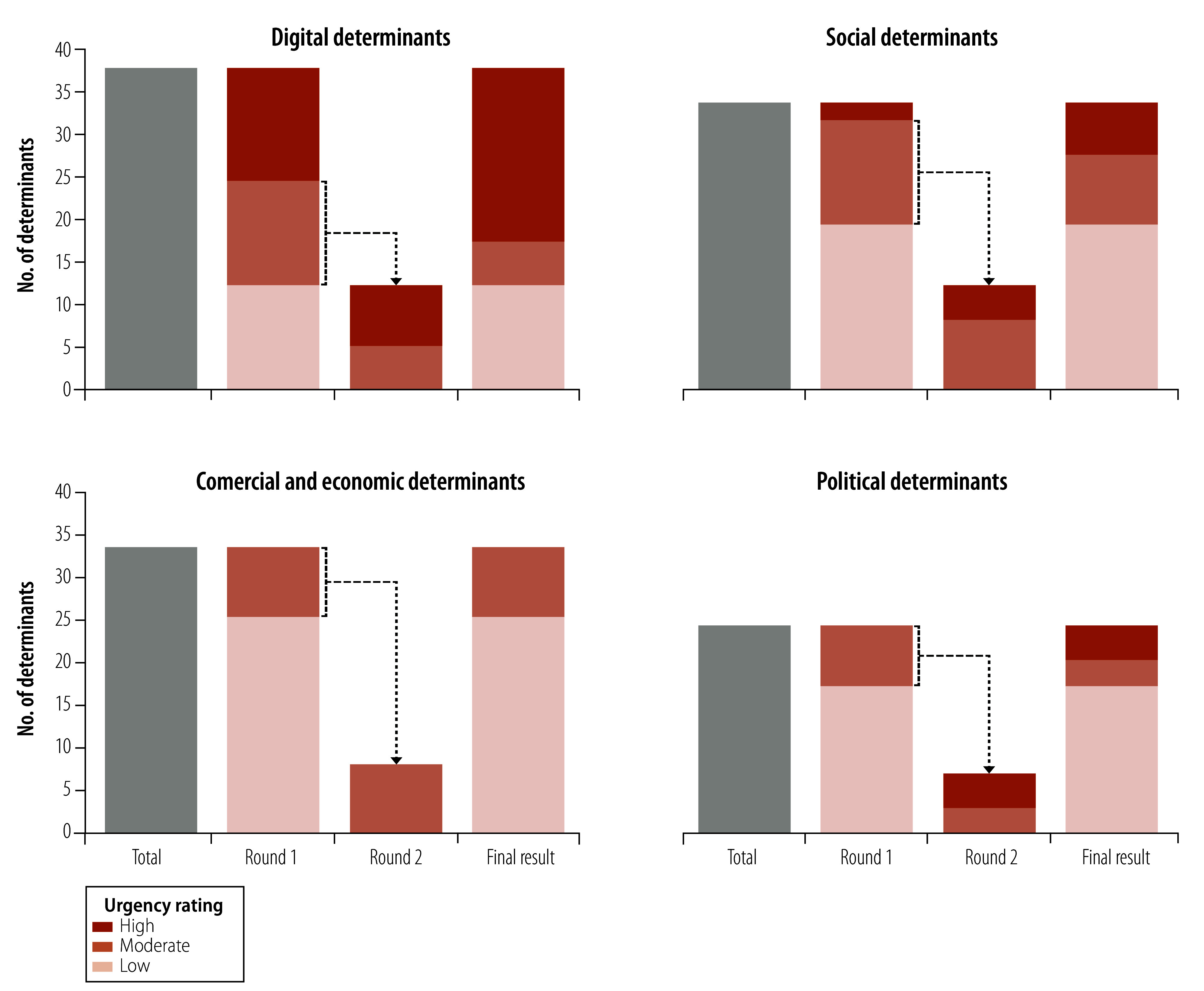
Urgency rating of health determinants in the digital age, by health determinant domain

Note: Authors and contributors rated health determinants identified through a scoping review in two rounds. In the first round, all 127 identified determinants were rated as high, moderate or low. In the second round, only determinants rated as moderate in first round were re-evaluated.

Of the 54 authors and contributors, 35 (64.8%) fully completed the first survey round and 32 (59.3%) fully completed the second round. After Round 1, consensus was reached for 88 out of 127 determinants, leaving 39 determinants to be re-rated in Round 2. Ultimately, by consensus, the survey panel considered 30 determinants (23.6%; 20 digital, 6 social, 0 commercial and economic, 4 political) as highly urgent (Table 2; Fig. 3; online repository).^36^

**Table 2:** High-urgency health determinants in a digital age ranked by consensus

Digital ecosystem domain, societal category, health determinant	Urgency			
	Median (IQR)	% (no. of respondents/total respondents)^a^	Consensus round	
**Digital domain** *Person-specific determinants*			
Internet access and connectivity	5 (1)	86.11 (31/36)	Round 1	
Device and software availability	4 (1)	81.82 (27/33)	Round 2	
Digital literacy	4 (1)	80.56 (29/36)	Round 1	
*Community determinants*			
Data and digital capacity	4 (1)	80.00 (28/35)	Round 1	
*Technology-related determinants*			
Moderation of harmful content and misinformation	4 (1)	88.57 (31/35)	Round 1	
Model accuracy and algorithmic validation	5 (1)	80.00 (28/35)	Round 1	
Data and digital interoperability	5 (1)	91.43 (32/35)	Round 1	
Explainability	4 (1)	93.75 (30/32)	Round 2	
Security settings and features	4 (1)	90.62 (29/32)	Round 2	
*Policy determinants*			
AI validation, transparency, explainability, accountability and ethics	5 (1)	91.43 (32/35)	Round 1	
Privacy and security policy	4 (1)	80.00 (28/35)	Round 1	
Access and sharing policy	4 (1)	82.86 (29/35)	Round 1	
Data consent policy	5 (1)	87.50 (28/32)	Round 2	
Mis- and/or disinformation policy	4 (1)	80.00 (28/35)	Round 1	
Outcomes, utility and value sharing	4 (1)	84.38 (27/32)	Round 2	
*Political, economic, societal and cultural determinants*			
Digital divides	4 (1)	94.29 (33/35)	Round 1	
Data governance and ethics	5 (1)	85.71 (30/35)	Round 1	
Data culture	4 (1)	81.25 (26/32)	Round 2	
Regulatory mandate	4 (0)	90.62 (29/32)	Round 2	
Digital public infrastructure	4 (1)	91.43 (32/35)	Round 1	
**Social domain** *Person-specific determinants*			
Access to health and social services	5 (1)	81.08 (30/37)	Round 1	
Health and disability status	4 (1)	93.94 (31/33)	Round 2	
*Technology-related determinants*			
Inclusive design	4 (1)	81.25 (26/32)	Round 2	
Good practice design	4 (0)	87.50 (28/32)	Round 2	
*Policy determinants*			
Health and social care policy	5 (1)	85.71 (30/35)	Round 1	
*Political, economic, societal and cultural determinants*			
Socioeconomic inequalities	4 (0)	90.62 (29/32)	Round 2	
**Political domain** *Community determinants*			
Ownership of technology	4 (0)	81.25 (26/32)	Round 2	
*Political, economic, societal and cultural determinants*			
Digitalization agenda	4 (0)	84.38 (27/32)	Round 2	
Accountability and transparency of commercial interests	5 (1)	84.38 (27/32)	Round 2	
Open and transparent decision-making	4 (1)	93.75 (30/32)	Round 2	

IQR: interquartile range.

^a^ Urgency percentage was defined as the combined proportion of 4 (urgent) and 5 (very urgent) ratings. Incomplete responses were included in these results.

## Discussion

Here we identified 127 determinants that can affect health directly or indirectly through the digital world. The distribution of determinants was relatively balanced across the different domains, underscoring the transformative impact of digitalization on health. The accompanying conceptual framework highlights that the influence of the domains of determinants does not occur in isolation but rather in combination across multiple parts of society.

Our findings reinforce the importance of ensuring that digital transformations are equitable and sustainable.^38^^,^^55^ While younger and healthier populations are better equipped to use digital tools, they are also more likely to be exposed to the adverse effects of digital transformations.^8^^,^^17^ In contrast, people most likely of digital exclusion, such as older people, people living with disabilities or higher disease burden, migrants or other vulnerable groups, may gain the most from digital health-care transformations, but are also among the best protected from its adverse effects.^55^^,^^181^ Various solutions have been suggested to address this digital health paradox, including improving digital access and literacy of vulnerable populations, and placing them at the centre of the digital health design process.^23^ Furthermore, we must consider how the digital divides evolve over time. The uneven introduction of basic information technologies, such as mobile phones and computers, creates digital divide as access to and engagement with these technologies directly affect participation in the digital society and health system.^99^^,^^182^ Subsequently, the introduction of more advanced digital technologies, such as artificial intelligence (AI), blockchain technology and spatial computing, may trigger a second digital divide. A key difference between these two divides is that the second divide also includes elements of the first, resulting in a more complex and heterogenous digital divide. Furthermore, the projected increase in socioeconomic inequalities over the next 30 years may also worsen the digital divide.^9^^,^^183^ Therefore, continuous monitoring, review and adaptation of policies and practice related to digital (health) technologies will remain important.

The identified determinants highlight how pervasive digital content can be sustained through the intersection of digital, commercial and economic and political determinants at multiple levels of governance.^93^^,^^155^^,^^184^ For example, dark commercial patterns, that is digital choice architecture that subvert consumer decision-making, have been rooted at the centre of the design and implementation of commercial digital solutions, contributing to problematic internet use, cyberbullying, hostile communication or peer activity, online sexual harassment, non-consensual messaging and building user communities aimed at harmful behaviour.^8^^,^^42^^,^^43^^,^^93^^,^^155^ Similarly, although major technological companies have reportedly disabled targeted advertisement towards minors, children can still be exposed to digital marketing through shared household devices, especially in areas with lower internet access or fewer devices.^6^^,^^8^^,^^9^

Compared to other sectors, the health sector is in a unique position in digitalization and individual data rights, as health data are considered sensitive,^185^^–^^187^ especially for people with diminished autonomy, such as minors and people living with certain disabilities. This position requires higher standard and security measures for responsible data use.^188^ Simultaneously, the (re)use of health data is important for scientific progress in public health, medicine and population health management. To fully realize the benefit of these sensitive data, national legal frameworks need to allow the secondary use of health data,^88^^,^^187^ while adhering to existing principles for equitable health data governance.^188^^,^^189^ Policy-makers linked to the health sector must, therefore, be educated and empowered to keep up with advances in privacy and security technologies.^93^

Furthermore, developing robust data and digital governance policies requires a profound understanding of the underlying normative and ethical principles, which can substantially differ between countries and global regions.^190^^–^^195^ For example, data governance in Europe, Canada and the United States of America is influenced by Kantian principles, which emphasize people's ability to retain control over their data, and that personal data cannot be used for secondary purposes without explicit consent.^192^ This approach prioritizes individual privacy and safety.^193^^,^^194^ In contrast, data governance approaches found in the South-East Asia and Western Pacific regions are influenced by Confucian principles.^190^^,^^191^ While these principles emphasize respect for individual autonomy, they place greater importance on the interests of the family and the community over those of the individual,^190^ thereby promoting collectivism while still respecting individual integrity. However, local data governance policies reflecting local norms and values may not always apply to health data of the local population, especially with global service providers. For example, data may be transferred to the country where servers are located, allowing the host country's policies to override those of the data subject’s country.

Finally, the widespread emergence of AI technologies in recent years has highlighted both the potential benefits^196^^–^^204^ and risks with its use, especially if its capabilities are deployed for other interests than the public good.^9^^,^^62^^,^^162^^,^^197^^,^^205^^–^^210^ Recognizing the impact of AI technologies on the political and commercial and economic domains is crucial, as both can have possible repercussions for individual and population health and well-being.^9^^,^^162^^,^^197^^,^^206^ Simultaneously, benefits and risks of AI technologies are unevenly spread across different population groups.^210^^–^^212^ While AI is categorized under the digital determinants of health as a sector-specific policy, a single set of AI policies is unlikely to suffice, especially recognizing the vastly different ways in which AI affects various sectors. As different archetypes of AI technologies are introduced in society, policy-makers must implement discipline- and archetype-specific policies to complement baseline regulations that address AI holistically, for example the AI Act in the European Union.^213^ A baseline policy framework can serve as an important foundation for developing more discipline-specific regulation,^214^ as it can address widespread risks of AI, for example by creating a risk framework to mitigate unacceptable risks to citizens and providing guidelines for high- or low-risk AI technologies.

While the presented framework is primarily intended to help decision-makers to identify the broad range of pathways that can affect individual and population health, it is also valuable to technology developers and decision-makers outside the health field. The framework can raise awareness among non-health experts about how their decisions can influence individual and population health, especially considering both the positive and negative influences of digital transformations. In doing so, the framework provides a more balanced and comprehensive view of digital transformation, compared to many articles which focus solely on either the beneficial or harmful aspects. This conceptual framework thus complements existing frameworks covering pathways for implementing digital health applications,^23^^,^^160^ while also informing targeted action to mitigate or prevent the harmful effects of digitalization on society.^8^^,^^69^^,^^155^^,^^177^ The conceptual framework also combines two competing interpretations of the position of the digital determinants of health within the landscape of health determinants. By recognizing that some social, commercial and economic and political determinants have changed during a period of rapid digital advancements, we support the idea that the effects of the digital world can be observed in how traditional determinants of health have adapted to digitalization.^10^^,^^38^^,^^81^^,^^215^ Simultaneously, our framework recognizes that certain health determinants did not exist before the rapid digital advancements, supporting the development of a separate category for digital determinants of health.^10^^,^^59^^,^^79^^,^^216^

This study has some limitations. First, the findings of this review should be interpreted as a high-level literature overview, and therefore potentially missing more intricate or localized factors related to health determinants affected by rapid digital advancements and proliferation in society. Second, selection bias is possible, as only three academic databases and Google Scholar were used, and the search strategy was not exhaustive. Third, the quality of the included sources was not assessed, which should be considered when interpreting the results. However, as this study aimed to assess changes in health determinants during rapid digital advancements, rather than validate methodological rigour, the absence of a quality assessment does not undermine the validity of this study. In fact, the information collected did not solely rely on scientific articles, as our international and multidisciplinary author and collaborator team also ensured that the collected information was comprehensive, accurate and unbiased across global regions, strengthening our confidence in the potential broad applicability of these findings to various countries and cultural settings. Finally, we acknowledge that this review makes broad conclusions about health determinants in a period of rapid digital advancements and the priority areas therein, which may not be directly transferable to localized contexts.

Our findings can inform future research exploring the interlinkages between digital, social, commercial and political factors to better understand their multifaceted effect on health. Additionally, the concept of health determinants during rapid digital advancements and proliferation in society, especially the digital determinants of health, is likely to evolve into patterns that are not yet predictable. This change means that the digital determinants of health outlined in this article may need to be redefined over time, as more research is conducted and emerging technologies become more integrated into health and social systems. This article can serve as a starting point for future research to monitor the developments of health determinants during rapid digital advancements. The findings also challenge us to better understand how health is affected by rapid digital advancements, and how determinants interact to facilitate the development, sustainability and improvement of the digital environment.
